# Evaluation and Validation of Commercially Available Dopamine Transporter Antibodies

**DOI:** 10.1523/ENEURO.0341-22.2023

**Published:** 2023-05-03

**Authors:** Emma E. Russo, Lola E. Zovko, Reza Nazari, Hendrik Steenland, Amy J. Ramsey, Ali Salahpour

**Affiliations:** Department of Pharmacology and Toxicology, University of Toronto, Toronto, Ontario M5S 1A8, Canada

**Keywords:** antibodies, dopamine transporter, immunoblotting, immunohistology, western blotting, SLC6A3

## Abstract

With a wide variety of dopamine transporter (DAT) antibodies available commercially, it is important to validate which antibodies provide sufficient immunodetection for reproducibility purpose and for accurate analysis of DAT levels and/or location. Commercially available DAT antibodies that are commonly used were tested in western blotting (WB) on wild-type (WT) and DAT-knock-out (DAT-KO) brain tissue and with immunohistology (IH) techniques against coronal slices of unilaterally lesioned 6-OHDA rats, in addition to wild-type and DAT-knock-out mice. DAT-KO mice and unilateral 6-OHDA lesions in rats were used as a negative control for DAT antibody specificity. Antibodies were tested at various concentrations and rated based on signal detection varying from no signal to optimal signal detection. Commonly used antibodies, including AB2231 and PT-22 524-1-AP, did not provide specific DAT signals in WB and IH. Although certain antibodies provided a good DAT signal, such as SC-32258, D6944, and MA5-24796, they also presented nonspecific bands in WB. Many DAT antibodies did not detect the DAT as advertised, and this characterization of DAT antibodies may provide a guide for immunodetection of DAT for molecular studies.

## Significance Statement

Dopamine is an essential neurotransmitter that is involved in mood regulation, voluntary movement, motivation, reward, and sleep. The dopamine transporter (DAT) is responsible for the reuptake of dopamine, and is implicated in Parkinson’s disease, schizophrenia, substance use disorder, and depression, among other neuropsychiatric disorders. There are numerous commercially available antibodies that serve to detect DAT. Detection may be variable across these antibodies and their batch numbers, and proper detection of DAT is vital to understanding its protein levels or absence, as well as determining its location. In this study, we tested various DAT antibodies in mouse and rat brain tissues to assess and categorize their immunodetection of DAT.

## Introduction

Dopamine is a key neurotransmitter involved in the regulation of many functions including mood, voluntary movement, reward, sleep, and motivation (Torres, 2006; Schultz, 2007; Salamone and Correa, 2012). The dopamine transporter (DAT), a transmembrane protein found on presynaptic dopamine neurons, plays a crucial role in maintaining dopamine homeostasis through the reuptake of dopamine (Gainetdinov et al., 1998; Nelson, 1998; Gainetdinov and Caron, 2003; Torres et al., 2003b). Immunodetection of DAT is vital in dopamine-related research areas such as Parkinson’s disease (PD), schizophrenia, substance use disorder, attention-deficit-hyperactivity disorder (ADHD), depression, and other neuropsychiatric conditions. However, it is sometimes difficult to generate antibodies for membrane proteins such as DAT because of their complex structures and highly conserved sequences among species, leading to low immunogenicity and antibody production (Hashimoto et al., 2018).

In an effort to catalog and validate available and commonly used commercial DAT antibodies, we assessed the ability, or lack thereof, of various commercial DAT antibodies to provide satisfactory immunodetection in western blotting (WB) and immunohistology (IH). These efforts are aimed to contribute to and strengthen scientific reproducibility, as immunodetection of DAT allows for analysis of DAT protein levels, its presence or absence, and assessment of its location (Miller et al., 1997; Wilson et al., 1996; Melikian and Buckley, 1999). In essence, proper and sufficient immunodetection of DAT can be one of the critical aspects for studies involving the dopamine system and validated antibodies would be helpful for these efforts.

## Materials and Methods

### Animals

#### Rats

RNU nude rats were purchased from Charles River and permitted two weeks to recover before surgery. Rats were housed individually with a 12/12 h light/dark cycle and provided food and water *ad libitum*. On the day of surgery, the weight of the rats was between 193 and 234 g, with a mean of 213.5 g. Procedures conformed to the recommendations of the Canadian Council on Animal Care and the National Institutes of Health guidelines for the care and use of animals, with all animal procedures performed in accordance with the University of Toronto animal care committee’s regulations. In total, nine nude rats were used for the study (*N* = 4 males, *N* = 5 females).

#### Mice

For mouse experiments, wild-type (WT) and dopamine transporter knock-out (DAT-KO) mice (13–17 weeks old) with a C57BL/6J genetic background were used. Mice were housed on a 12/12 h light/dark cycle and food and water was provided *ad libitum*. Procedures conformed to the recommendations of the Canadian Council on Animal Care and the National Institutes of Health guidelines for the care and use of animals, with all animal procedures performed in accordance with the University of Toronto animal care committee’s regulations. Experiments were conducted using C57 black background-matched WT and DAT-KO littermate mice (Giros et al., 1996; Vecchio et al., 2014). Antibodies were verified on three independent samples at three separate times. In total, 10 WT (*N* = 5 males, *N* = 5 females) and 10 DAT-KO (*N* = 5 males, *N* = 5 females) mice were used for the study.

### Western blotting on whole-tissue lysate

Western blotting on whole tissue lysate was performed to quantify DAT protein expression. Briefly, the striatum was dissected from freshly harvested brains using a chilled surgical plate and dissection tools. Brain samples were mechanically homogenized using a VWR Disposable Pellet Mixer (47747-370) in RIPA buffer (50 mm Tris-HCl, pH 7.4, 100 mm NaCl, 1% Nonidet P-40, 0.5% sodium deoxycholate, 0.1% SDS; Sigma), 10 μg/ml of protease inhibitor leupeptin (Bioshop, LEU001.50), 5 μg/μl pepstatin A (Bioshop, PEP605), 1.5 μg/ml aprotinin (Bioshop, APR600), 0.1 μg/ml benzamidine (Bioshop, BEN601), 100 μm PMSF (Bioshop, PMS123), 2.5 mm Na pyro-phosphate (Bioshop, SPP310), 1 mm β-glycerophosphate (Bioshop, GYP001), 10 mm NaF (Bioshop, SFL001), and 1 mm Na_3_VO_4_ (Bioshop SOV664). Protein concentrations were measured using a BCA protein assay kit (Thermo Scientific, 23225). Proteins were separated on either a Novex 4–20% Tris-Glycine (Invitrogen, XP04205BOX), Novex 10% Tris-Glycine (Invitrogen, XP00100PK2), or homemade 10% Tris-Glycin acrylamide gels (Table 1). The total protein loaded was 30 μg per sample. Proteins were transferred onto a PVDF membrane (Pall Life Sciences, BSP161) for 90 min at 90V at room temperature (90v-90m-RT) or 22 V overnight at 4° (22v-ON-4; Table 1). Total protein loading was measured either using GAPDH antibody staining or using a LI-COR Total Protein Stain (LI-COR, LIC-926-11010; data not shown). Membranes were blocked with a LI-COR blocking buffer (LI-COR, LIC-927-40100) for 1–2 h at RT on a rocker and immuno-stained overnight at 4°C with primary antibodies (Table 1). After washing, protein bands were visualized on a high-resolution LI-COR imaging machine with fluorescence-labeled secondary antibodies donkey anti-rat IRdye 800 CW, goat anti-rabbit IRdye 800 CW, goat anti-mouse IRdye 680 or donkey anti-goat IRdye 680 CW (1:15,000 dilution; Rockland 612-731-120, LI-COR 926-32211, LI-COR 925-68074, respectively). Method details for each antibody are specified in Table 1.

**Table 1:** Specific methods used for each antibody for western blotting

Antibody	Gel	Protein	Concentration	Transfer	Blocking	Antibody buffer
MAB369; Millipore Sigma (lot #3188610)	Novex 4–20% Tris-Glycine	Mouse striatum total lysate	1:750	90v-90m-RT	LI-COR for 2 h at room temperature	LI-COR
600-401-C75; Rockland (lot #27999)	Novex 4–20% Tris-Glycine	Mouse striatum total lysate	1:1000	90v-90m-RT	LI-COR for 2 h at room temperature	LI-COR
AB2231; Sigma-Aldrich (lot #NG1900345)	Novex 4–20% Tris-Glycine	Mouse striatum total lysate	1:1000	90v-90m-RT	LI-COR for 2 h at room temperature	LI-COR
SC-1433; Santa Cruz (lot #B1413)	Novex 10% Tris-Glycine	Mouse striatum total lysate	1:750	90v-90m-RT	LI-COR for 2 h at room temperature	LI-COR
SC-32259; Santa Cruz (lot #l1021; l2717)	Novex 10% Tris-Glycine	Mouse striatum total lysate	1:750	90v-90m-RT	LI-COR for 2 h at room temperature	LI-COR
SC-32258; Santa Cruz (lot #F0722; G1217)	Novex 10% Tris-Glycine	Mouse striatum total lysate	1:750	90v-90m-RT	LI-COR for 2 h at room temperature	LI-COR
SC-58517; Santa Cruz (lot #C2216)	Novex 10% Tris-Glycine	Mouse striatum total lysate	1:750	90v-90m-RT	LI-COR for 2 h at room temperature	LI-COR
431-DATC; Phospho-Solutions (lot #ajo1016y)	Homemade 10% Tris-Glycin acrylamide	Mouse striatum total lysate or cell total lysate	1:1000	90v-90m-RT	LI-COR for 2 h at room temperature	LI-COR
434-DATEL2; Phospho-Solutions (lot #jh417g)	Novex 10% Tris-Glycine	Mouse striatum total lysate	1:1000	90v-90m-RT	LI-COR for 2 h at room temperature	LI-COR
22524-1-AP; Thermo Fischer Scientific (lot #000025322)	Homemade 10% Tris-Glycin acrylamide	Mouse striatum total lysate or cell total lysate	1:1000	90v-90m-RT	LI-COR for 1.5 h at room temperature	LI-COR
GTX30992; Gene Tex (lot #821800340)	Homemade 10% Tris-Glycin acrylamide	Mouse striatum total lysate	1:500	90v-90m-RT	LI-COR for 1.5 h at room temperature	LI-COR
D6944; Millipore Sigma (lot #000018709; 069M4849V)	Homemade 10% Tris-Glycin acrylamide	Mouse striatum total lysate	1:500	90v-90m-RT	LI-COR for 1.5 h at room temperature	5% BSA in TBS+ 0.05% Tween 20
MA5-24796; Thermo Fisher Scientific (lot #XF3606356)	Novex 10% Tris-Glycine	Mouse striatum membrane preparation	1:250	22v-ON-4	5% BSA in TBS for 1 h at room temperature	5% BSA In TBS+ 0.05% Tween 20
ZRB1525; Millipore Sigma (lot #Q3480251; Q3451217)	Novex 10% Tris-Glycine	Mouse striatum membrane preparation	1:100	22v-ON-4	5% BSA in TBS for 1 h at room temperature	5% BSA In TBS+ 0.05% Tween 20

### Western blotting on membrane preparation samples

For antibodies ZRB1525 and MA5-24796, western blotting using membrane preparation was performed to further enrich for DAT protein, as DAT is a membrane protein. All work was done on ice. Striatal dissected samples were transferred to a 25 mm Tris-2 mM EDTA solution with protease inhibitors 10 μg/ml leupeptin (Bioshop, LEU001.50), 5 μg/μl pepstatin A (Bioshop, PEP605), 1.5 μg/ml aprotinin (Bioshop, APR600), 0.1 μg/ml benzamidine (Bioshop, BEN601), 100 μm PMSF (Bioshop, PMS123), 2.5 mm Na pyro-phosphate (Bioshop, SPP310), 1 mm β-glycerophosphate (Bioshop, GYP001), 10 mm NaF (Bioshop, SFL001), and 1 mm Na_3_VO_4_ (Bioshop SOV664) and homogenized using a Polytron Tissue Homogenizer (47751-624). Samples were then centrifuged at 600 × *g* for 10 min at 4°C. The supernatant was then transferred to a thick-wall Sorvall tube (Thermo Fischer Scientific) and spun at 40,000 × *g* for 20 min at 4°C (Sorvall centrifuge, Thermo Fischer Scientific). The supernatant was discarded, and the pellet resuspended in the Tris-EDTA+inhibitors solution. Protein concentration was measured using a BCA assay (Thermo Scientific, 23225) and subsequent western blotting (as previously mentioned) was performed.

### Western blotting using HEK293 cells

Methodology for HEK293 expressing YFP-HA-βLAC-DAT construct, transfection, and generation of cell lines used was described previously (Beerepoot et al., 2016; Sutton et al., 2022). Briefly, HEK293 cells were maintained in DMEM (Sigma) supplemented with 10% FBS (Sigma), 100 U/ml penicillin and 100 μg/ml streptomycin. Cells were kept in 5% atmospheric CO_2_ and 37°C. Cells expressing YFP-HA-βLAC-DAT were further supplemented with 1 μg/ml puromycin. Stable cell lines expressing the YFP-HA-βLAC-DAT were lysed in RIPA buffer supplemented with protease inhibitors 10 μg/ml leupeptin (Bioshop, LEU001.50), 5 μg/μl pepstatin A (Bioshop, PEP605), 1.5 μg/ml aprotinin (Bioshop, APR600), 0.1 μg/ml benzamidine (Bioshop, BEN601), 0.1 mm PMSF (Bioshop, PMS123), all on ice. At 4°C, cell lysates were shaken for 15 min followed by centrifugation at 15,000 rpm for 15 min to pellet debris. Protein concentrations were measured using a BCA protein assay kit (Thermo Scientific, 23225) and subsequent western blotting (as previously mentioned) was performed.

### 6-OHDA microinjection surgery

Under the CCAC animal care recommendations for the reduction of animal use, we decided reuse control RNU nude rats from a prior study. In that study we unilaterally lesioned the dopamine neurons via a 6-OHDA injection in the medial forebrain bundle. Nude rats were required in this prior study because the experimental (but not control group) required human stem cell implantation. Surgeries and injections were conducted in sterile conditions in a biosafety cabinet. The control group of rats (no cell implant) received a 6-OHDA lesion in the medial forebrain using procedures described elsewhere (Kirik et al., 1998). In brief, rats were induced with anesthesia with 5% isoflurane and maintained with ∼2% isoflurane while secured to a stereotaxic frame (Kopf Instruments, Model 963). The stereotaxic coordinates were calculated and adjusted for each animal for 6-OHDA injection (relative to bregma, AP = −3.9, ML = 1.2). At the correct coordinates, 0.8 mm holes were drilled in the skull overlying the right medial forebrain bundle. A 10 μl Hamilton syringe (Hamilton Company) was placed in the pump with a 2-inch, 33-G needle, with a 45° tip angle (Hamilton Company). Hamilton syringe pumps were loaded with newly thawed (within 1 min while covered) 6-OHDA (Sigma-Aldrich) solution which was prepared at 8 μg/μl in 0.9% sterile saline solution containing 0.02–0.04% of the antioxidant L-ascorbic acid. Hamilton syringe needles were directed to the medial forebrain bundle (from dura, DV = 7.7) and 6-OHDA was microinjected (20 μg, 2.5 μl volume, 0.5 μl/min). Five minutes following the completion of microinjection, the needle was raised by 0.25 mm. One minute later, the needle was slowly and completely retracted from the brain. The scalp was sutured with an absorbable suture. Animals were monitored continuously to ensure their health and wellbeing postsurgery. After 36 weeks, a total of nine rats were euthanized after 6-OHDA treatment and used for histologic studies.

### Immunohistology

#### LI-COR Immunohistology

DAT and TH immunohistology (IH) protocol used was performed as described previously (Vecchio et al., 2014; Masoud et al., 2015). 6-OHDA rat brains were perfused via cardiac perfusion with 4% paraformaldehyde (PFA) then removed and stored in 4% PFA for 24 h followed by 10% sucrose for at least 48 h. Mouse WT and DAT-KO brains were removed after cervical dislocation and placed into 4% PFA for 24 h followed by 10% sucrose for at least 48 h (Vecchio et al., 2014). Briefly, IH was performed on coronal sections 40 μm thick, cut from the brains of adult WT and DAT-KO mice and 6-OHDA unilateral lesioned rats. Coronal brain slices were mounted on slides bordered with a hydrophobic pen (Gnomepen, Invignome) and quenched with 0.5% sodium borohydride in PBS. Sections were then blocked for 2 h at room temperature [10% normal goat serum (NGS)], 0.75% bovine serum albumin (BSA; 0.1% Triton X-100, in PBS). Blocking was followed by an overnight incubation at 4°C in solution containing anti-DAT antibody and blocking buffer (Table 2). Anti-TH staining (Table 2) was performed on rat slices to demonstrate the efficiency of the 6-OHDA lesion and as a positive control. Sections were then washed in chilled PBS and incubated with secondary antibodies (Table 2) for 1 h at room temperature and prepared for high resolution LI-COR imaging by coverslip mounting using Thermo Scientific Aqua-Mount Slide Mounting Media (Thermo Scientific, 143905). Slices were scanned at an offset of 0.9 mm. Method details for each antibody are specified in Table 2.

**Table 2:** Specific methods used for each antibody for immunohistology

Primary antibody	Concentration	Buffer	Secondary antibody	Concentration	Buffer
22524-1-AP, Thermo Fisher Scientific (lot #000025322)	1:100, 1:200	2% NGS, 0.01% Triton X-100	Anti-rabbit IRdye 680 (lot #C51104-05)	1:2000	2% NGS, 0.01% Triton X-100
AB2231, Sigma-Aldrich (lot #NG1900345)	1:100, 1:200	2% NGS, 0.01% Triton X-100	Anti-rabbit IRdye 680 (lot #C51104-05)	1:2000	2% NGS, 0.01% Triton X-100
SC-32258, Santa Cruz (lot #F0722; G1217)	1:100, 1:200	2% NGS, 0.01% Triton X-100	Anti-rat IRdye 680 (lot #21321)	1:2000	2% NGS, 0.01% Triton X-100
SC-32259, Santa Cruz (lot #l1021; l2717)	1:100, 1:200	2% NGS, 0.01% Triton X-100	Anti-rat IRdye 680 (lot #21321)	1:2000	2% NGS, 0.01% Triton X-100
SC-58517, Santa Cruz (lot #C2216)	1:100, 1:200	2% NGS, 0.01% Triton X-100	Anti-rat IRdye 680 (lot #21321)	1:2000	2% NGS, 0.01% Triton X-100
MAB369, Millipore Sigma (lot #3188610)	1:100, 1:200	2% NGS, 0.01% Triton X-100	Anti-rat IRdye 680 (lot #21321)	1:2000	2% NGS, 0.01% Triton X-100
ZRB1525, Millipore Sigma (lot #Q3480251; Q3451217)	1:100, 1:200	2% NGS, 0.01% Triton X-100; 2% NGS	Anti-rabbit IRdye 680, anti-rabbit Alexa Fluor 568 dye (lot #C51104-05;927620)	1:2000; 1:500	2% NGS, 0.01% Triton X-100, 2% NGS
MA5-24796, Thermo Fisher Scientific (lot #XF3606356)	1:100, 1:200	2% NGS, 0.01% Triton X-100	Anti-mouse IRdye 680, anti-mouse Alexa Fluor 488 (lot #C50721-05;1110070)	1:2000; 1:500	2% NGS, 0.01% Triton X-100, 2% NGS
431-DATC, Phospho- Solutions (lot #ajo1016y)	1:100, 1:200	2% NGS, 0.01% Triton X-100	Anti-rabbit IRdye 680 (lot #C51104-05)	1:2000	2% NGS, 0.01% Triton X-100
D6944, Millipore Sigma (lot #000018709; 069M4849V)	1:200	2% NGS, 0.01% Triton X-100; 2% NGS (FM)	Anti-rabbit IRdye 680, anti-rabbit Alexa Fluor 568 dye (lot #C51104-05;927620)	1:2000; 1:500	2% NGS, 0.01% Triton X-100, 2% NGS
Anti-TH Antibody Pel Freez (P40101-150)	1:500 (rat TH verification)	2% NGS, 0.01% Triton X-100	Anti-rabbit IRdye 680 (lot #C51104-05)	1:2000	2% NGS, 0.01% Triton X-100

### High-magnification immunohistology

The IH protocol for high-magnification immunohistology was adapted from methods described elsewhere (Vecchio et al., 2014). Coronal sections 40 μm thick were cut from the brains of adult WT and DAT-KO mice. Sections were placed in a 24-well plate and washed with 1.2% Triton X-100 in PBS. Sections were then blocked for one hour at room temperature [10% normal goat serum (NGS)]. Blocking was followed by an overnight incubation at 4°C in solution containing anti-DAT antibody and blocking buffer (Table 2). After antibody solutions were removed, sections were washed in 0.2% Tween 20 in PBS and incubated with secondary antibodies for 1 h at room temperature (Table 2). After an additional wash, slices were imaged after coverslip mounting with Vecta-Shield Mounting Liquid containing DAPI (VECTASHIELD, H-1200-10). Slices were scanned on a Revolve four Hybrid Fluorescence Microscope (ECHO, 76490-302) at 10×, 20×, and 40× magnification.

### Statistical analysis

Statistical analysis was not included or required for the presentation of the following results as only representative data are shown. No quantitative analysis was completed on performed imaging studies.

## Results

### Western blotting using antibodies against N-terminal epitopes of DAT

**Figure 1.**
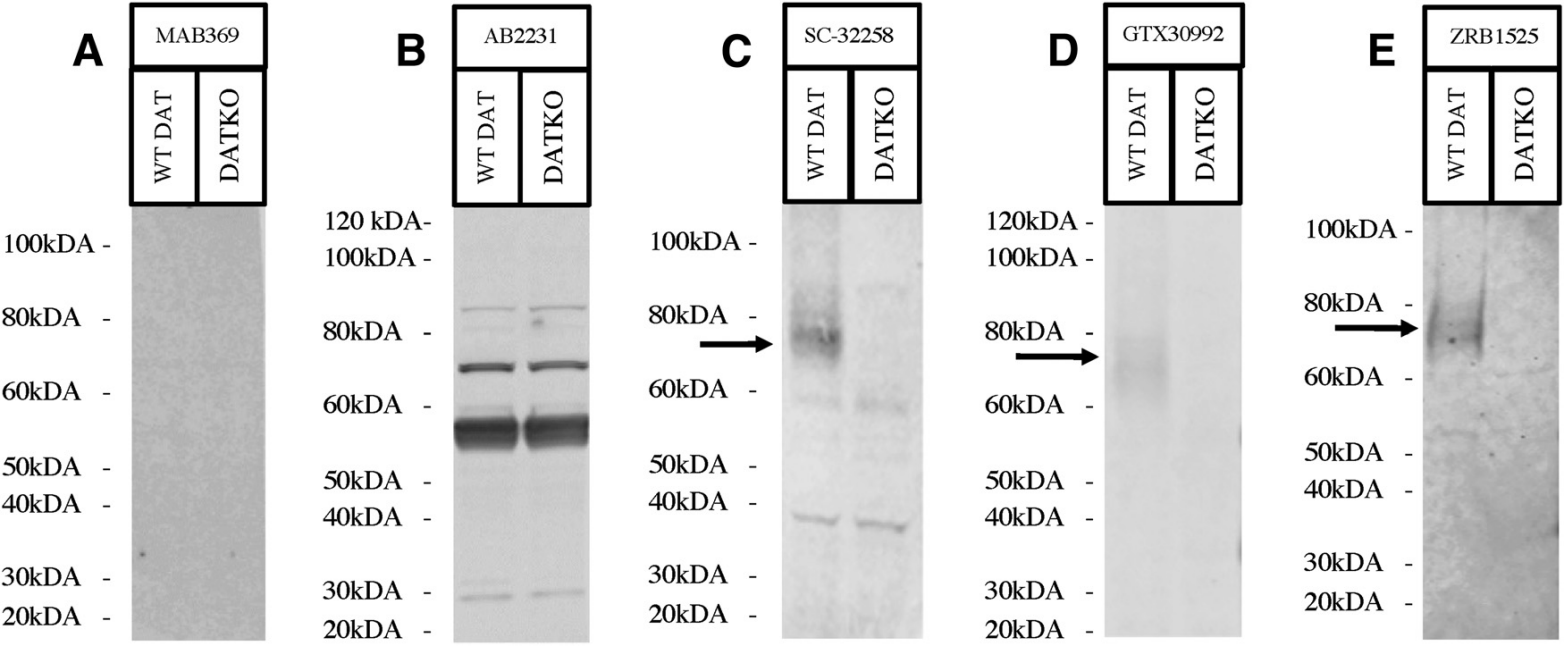
Western blots depicting anti-DAT antibodies with N-terminal immunogens. All western blots were performed using mouse striatum samples from DAT-knock-outs (DKO), and wild-type (WT) mice. Molecular ladder is indicated beside each figure, with detection of the dopamine transporter (DAT) indicated by a black arrow. Western blots include: (***A***) anti-DAT antibody MAB369 depicting no signal run on a Novex 4–20% Tris-Glycine gel using mouse striatum sample, (***B***) anti-DAT antibody AB2231 depicting strong nonspecific signal run on a Novex 4–20% Tris-Glycine gel using mouse striatum sample, (***C***) anti-DAT antibody SC-32258 depicting a moderate signal with some nonspecific bands run on a Novex 10% Tris-Glycine gel using mouse striatum sample, (***D***) anti-DAT antibody GTX30992 depicting a weak signal run on a homemade 10% Tris-Glycin acrylamide gel using mouse striatum sample, and (***E***) anti-DAT antibody ZRB1525 depicting a weak signal run on a Novex 10% Tris-Glycine gel using mouse striatum sample.

**Figure 2.**
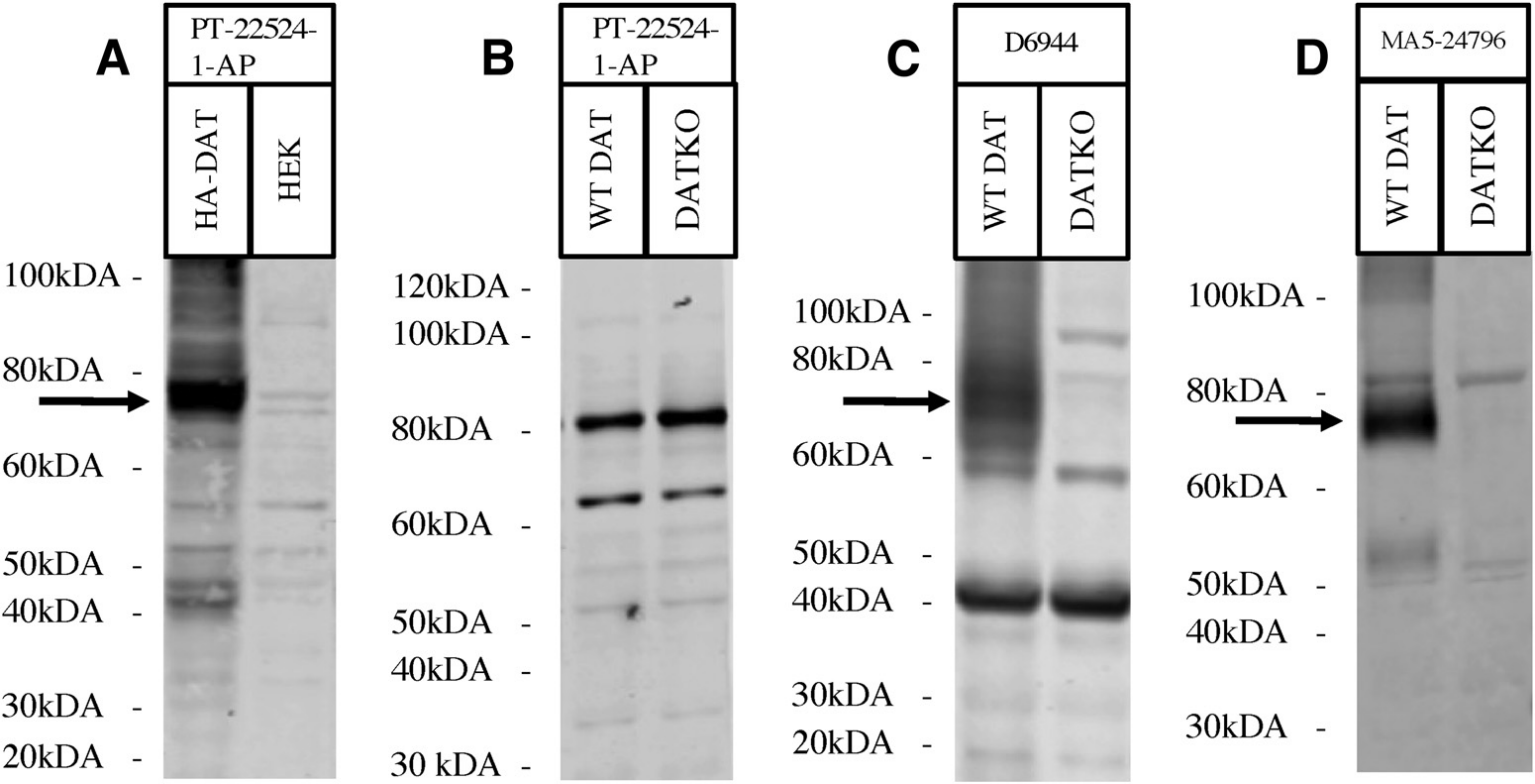
Western blots depicting the remaining anti-DAT antibodies with N-terminal immunogens. All western blots were performed using mouse striatum samples from DAT-knock-outs (DKO), and wild-type (WT) mice. Total cell lysates were also obtained and utilized from HEK293 and DAT-expressing HEK293 cells. Molecular ladder is indicated beside each figure, with detection of the dopamine transporter (DAT) indicated by a black arrow. Western blots include: (***A***) anti-DAT antibody PT-22524-1-AP depicting a strong signal with nonspecific binding on a homemade 10% Tris-Glycin acrylamide gel using total cell lysate, (***B***) anti-DAT antibody PT-22524-1-AP depicting strong nonspecific signal on a homemade 10% Tris-Glycin acrylamide gel using a mouse striatum sample, (***C***) anti-DAT antibody D6944 depicting a strong DAT signal with some nonspecific binding on a homemade 10% Tris-Glycin acrylamide gel using mouse striatum sample, and (***D***) anti-DAT antibody MA5-24795 depicting a strong DAT signal with minor nonspecific binding on a Novex 10% Tris-Glycine gel using mouse striatum sample.

Eight antibodies (MAB369, AB2231, SC-32 258, GTX30992, ZRB1525, PT-22524-1-AP, D6944, and MA5-24796) targeting the DAT N terminus were evaluated using western blotting. Under our conditions, antibody MAB369 showed no signal (Fig. 1*A*). Antibody AB2231 detects similar strong bands in WT and DAT-KO samples indicating that the detected bands are nonspecific and do not represent DAT (Fig. 1*B*). Antibody SC-32258 provided a moderate signal for DAT, did not display a DAT signal in the DKO sample, and showed the presence of some nonspecific bands (Fig. 1*C*). Antibody GTX30992 showed a weak signal for DAT that was not present in the DKO sample, with no nonspecific bands (Fig. 1*D*). Antibody ZRB1525 showed a weak signal for DAT that was not present in the DKO sample, and there were no nonspecific bands (Fig. 1*E*). Antibody PT-22524-1-AP showed good detection of DAT in HEK293 cells with the presence of some low-density nonspecific bands (Fig. 2*A*). However, PT-22524-1-AP did not demonstrate adequate detection of DAT in animal tissue (Fig. 2*B*) and only detected nonspecific bands in WT and DKO samples. Antibody D6944 had strong detection of DAT that was not present in the DKO sample, with the presence of several strong nonspecific bands (Fig. 2*C*). Antibody MA5-24796 displayed a very good detection of DAT that was not present in the DKO sample with some low-density nonspecific bands (Fig. 2*D*). It is important to note that the Western blotting of antibody ZRB1525 and MA5-24796 were performed on striatal membrane preparations. This was done to further enrich the membrane fraction which contains DAT and as expected it resulted in cleaner and better detection with these antibodies. Western blotting results are scored (Table 3) and summarized in Table 4.

**Table 3:** Score allocated to each antibody for detection of DAT in western blotting and immunohistology in rat and mouse tissue scale adapted from Lyck et al., 2008

Score	Classification	Definition
0	No signal	No specific signal
1	Poor signal	Very insufficient signal: presenting a very weak signal, false-negative signal of cells or animal tissue, or false-positive signal in cell or tissue samples.
2	Borderline signal	Insufficient signal: presenting too weak of a signal, false-negative signal or false-positive signal of cells or animal tissue.
3	Good signal	Signal is acceptable visualizing the appropriate protein band. Signal could still be optimized for improving the intensity and signal-to-noise ratio.
4	Optimal signal	Perfect or close to perfect signal result visualizing the appropriate protein in cells and tissue samples with low signal noise.

**Table 4:** Scale for scoring of western blotting and immunohistology results

Antibody	Western blotting score	Western blotting notes	Mouse IH score	Rat IH score	IH notes
MAB369; Millipore Sigma	0	No signal depicted at 1:750 dilution and very weak signal at 1:250 dilution (data not shown).	2	0	Moderate signal in mouse tissue. No signal in rat tissue.
AB2231; Sigma-Aldrich	0	No specific signal with several strong nonspecific bands.	0	0	No signal. Not detectible in rat.
SC-32258; Santa Cruz	2–3	Moderate signal with some weak nonspecific bands only at 1:750 but not 1:1000 (data not shown).	3	0	Good signal in mouse tissue. No signal in rat tissue.
GTX30992; Gene Tex	2	Weak signal, no discernable nonspecific band. *Antibody has been discontinued	n/a	n/a	
ZRB1525; Millipore Sigma	1–2	Weak DAT signal. No nonspecific bands.	4	3–4	Strong signal in mouse at 1:100 and 1:200. Good signal at high magnification. Good signal in rat at 1:100.
PT-22524-1-AP; Protein Tech	0 (mouse) 2 (cells)	Strong signal in cells with several nonspecific bands. No specific signal and many nonspecific bands in brain tissue.	1	0	Weak DAT signal with background/nonspecific signal in mouse brain. No signal in rat.
D6944; Millipore Sigma	3–4	Strong DAT signal with several strong nonspecific bands.	3	1	Good signal in mouse at 1:200. Good signal in mouse at high magnification. Weak signal in rat.
MA5-24796; Thermo Fisher Scientific	3–4	Very good DAT signal with some weak nonspecific bands.	3–4	1	Strong signal in mouse tissue. No signal in rat. Good signal at high magnification.
431-DATC; Phospho-Solutions	1	Weak signal with nonspecific bands in animal tissue.	2	0	Faint signal in mouse tissue. No signal in rat.
600-401-C75; Rockland	0	No signal.	n/a	n/a	
SC-1433; Santa Cruz	3–4	Good signal. Weak nonspecific bands. *Antibody has been discontinued	n/a	n/a	
SC-32259; Santa Cruz	0	No specific DAT signal with some weak nonspecific bands.	2	0	Only provided good signal detection in mouse tissue at 1:100. No signal in rat.
SC-58517; Santa Cruz	0	No signal.	1	0	Faint signal in mouse at 1:100 and 1:200 and no signal in rat tissue.
434-DATEL2; Phospho-Solutions	0	No specific signal with several strong nonspecific band.	n/a	n/a	

### Western blotting using antibodies against C-terminal epitopes on DAT

**Figure 3.**
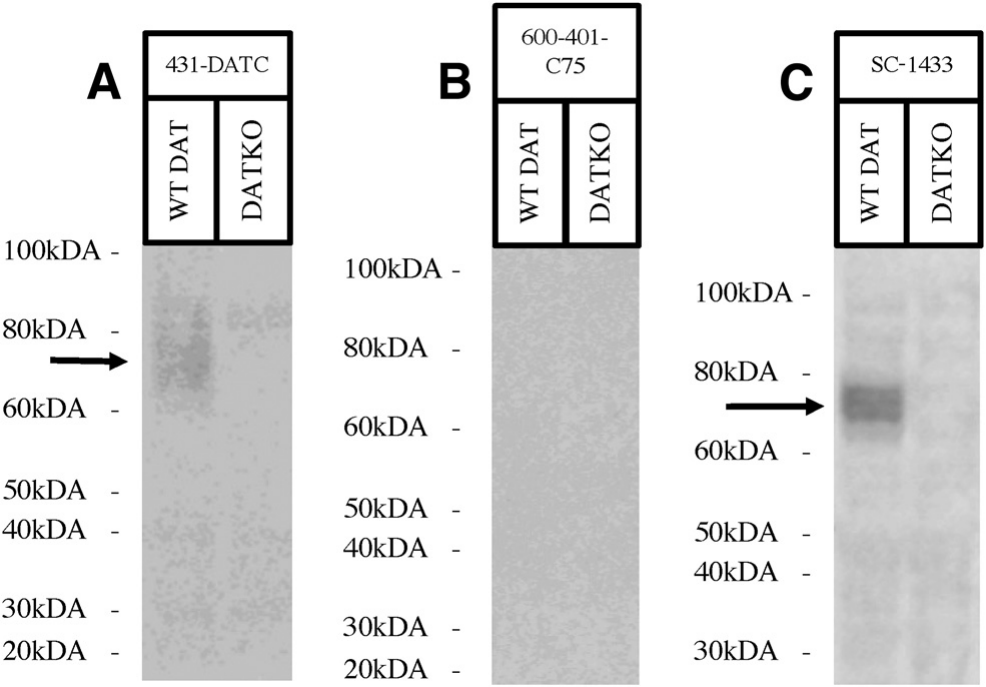
Western blots depicting anti-DAT antibodies with C-terminal immunogens. All western blots were performed using mouse striatum samples for wild-type (WT) and DAT-knock-outs (DKO) mice. Molecular ladder is indicated beside each figure, with detection of the dopamine transporter (DAT) indicated by a black arrow. Western blots include: (***A***) anti-DAT antibody 431-DATC depicting a moderate signal with some nonspecific binding on a homemade 10% Tris-Glycin acrylamide gel using mouse striatum sample, (***B***) 600-401-C75 depicting no signal run on a Novex 4–20% Tris-Glycine gel, and (***C***) SC-1433 depicting a strong DAT signal run on Novex 10% Tris-Glycine gel using mouse striatum sample.

Three antibodies (431-DATC, 600–401-C75, and SC-1433) targeting the DAT C terminus were evaluated using Western blotting. Antibody 431-DATC gave a weak DAT signal that was not present in the DKO sample, with some nonspecific bands (Fig. 3*A*). Antibody 600-401-C75 provided no signal (Fig. 3*B*). Antibody SC-1433 provided a good signal for DAT that was not present in the DKO sample, with very weak nonspecific bands (Fig. 3*C*). Western blotting results are scored (Table 3) and summarized in Table 4.

### Western blotting using antibodies against 2nd extracellular loop epitopes on DAT

**Figure 4.**
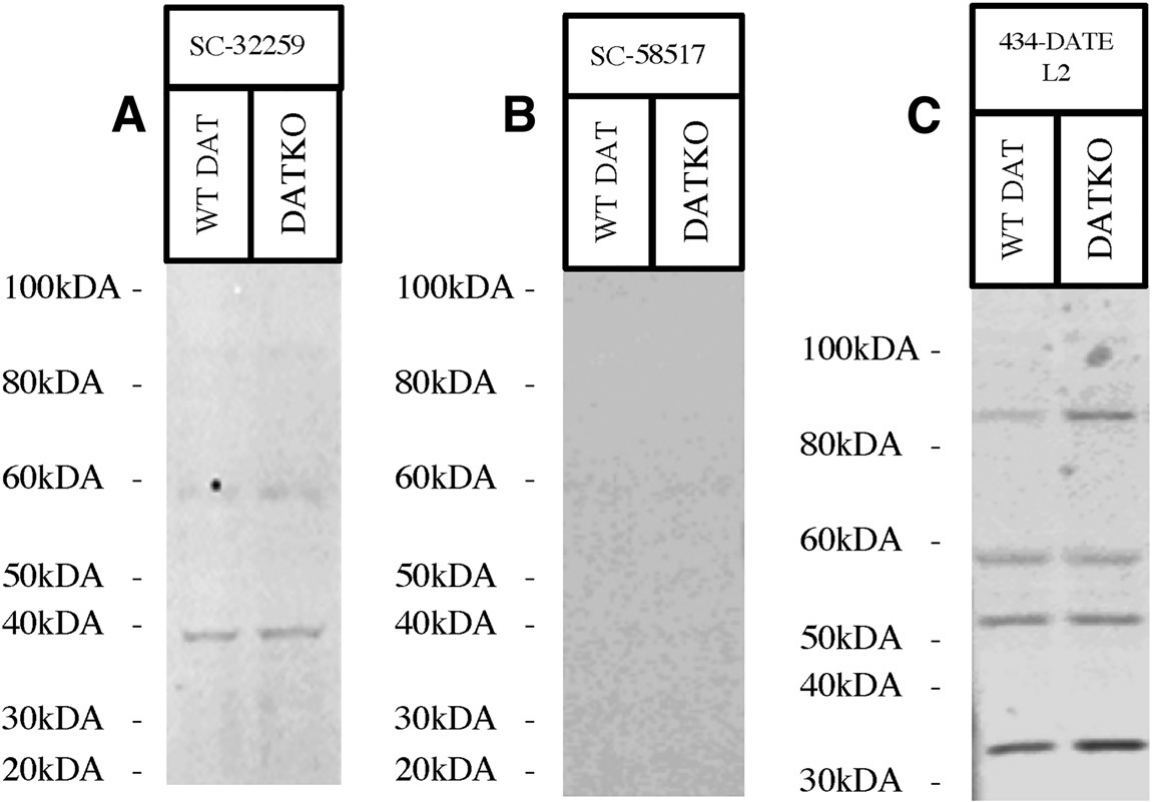
Western blots depicting anti-DAT antibodies with 2^nd^ extracellular loop immunogens. All western blots were performed using mouse striatum samples from DAT-knock-outs (DKO), and wild-type (WT) mice. Molecular ladder is indicated beside each figure, with detection of the dopamine transporter (DAT) indicated by a black arrow. Western blots using antibodies 2nd loop immunogens include: (***A***) anti-DAT antibody SC-32259 depicting no DAT signal and weak nonspecific binding on a Novex 10% Tris-Glycine gel using mouse striatum sample, (***B***) anti-DAT antibody SC-58517 depicting no DAT signal on a Novex 10% Tris-Glycine gel using mouse striatum sample, and (***C***) anti-DAT antibody 434-DATEL2 depicting no DAT signal and some strong nonspecific binding on a Novex 10% Tris-Glycine gel using mouse striatum sample.

Three antibodies (SC-32259, SC-58517, and 434-DATEL2) targeting the DAT 2nd extracellular loop were evaluated with western blotting. Antibody SC-32259 did not provide a DAT signal and produced some nonspecific bands (Fig. 4*A*). Antibody SC-58517 did not provide any signal (Fig. 4*B*). Antibody 434-DATEL2 did not provide a DAT signal and had several strong nonspecific bands (Fig. 4*C*). Western blotting results are scored (Table 3) and summarized in Table 4.

### Immunohistology using antibodies with DAT N-terminal immunogens

**Figure 5.**
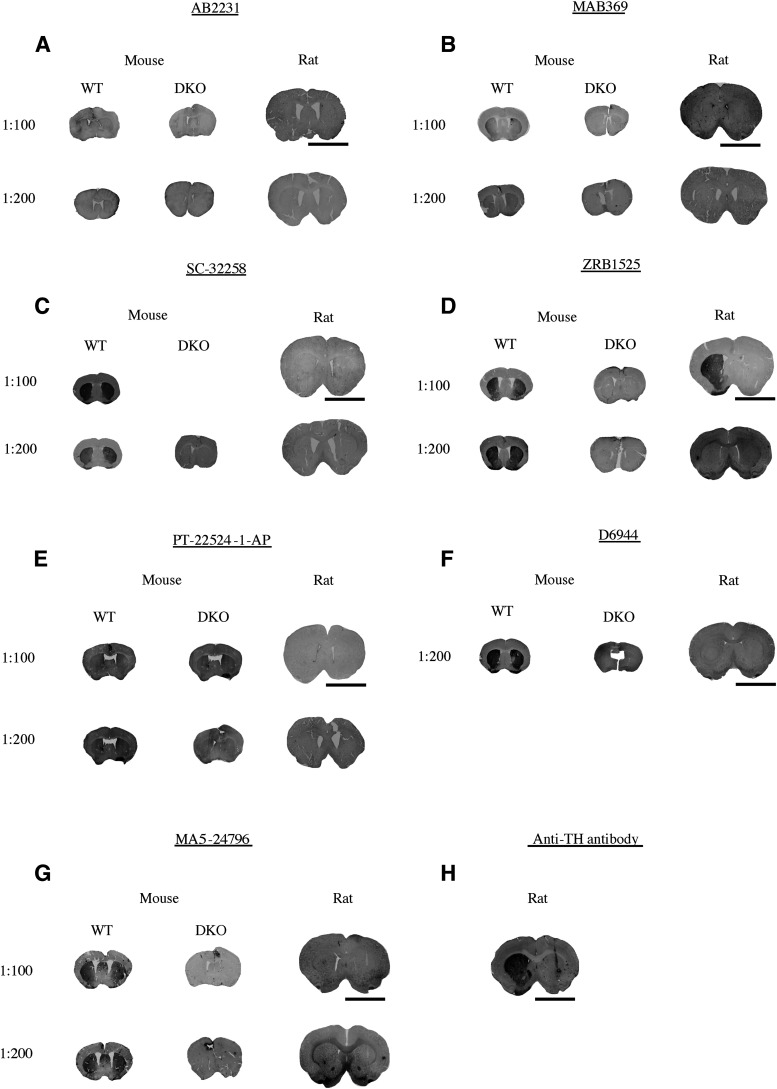
Immunohistology (IH) using antibodies with anti-DAT antibodies that have N-terminal immunogens. IH was performed using wild-type (WT) mouse tissue, DAT-knock-out (DKO) mouse tissue, or rat tissue in which one hemisphere was lesioned with 6-OHDA to kill dopamine neurons, thus eliminating the possibility of a DAT antibody to produce a signal on the lesioned side (internal negative control). Concentration of DAT antibody is indicated in the figure. ***A***, Anti-DAT antibody AB2231 displaying no signal in either mouse or rat tissue at a concentration of 1:100 or 1:200. ***B***, Anti-DAT antibody MAB369 displaying moderate signal in mouse tissue and no signal in rat tissue at a concentration of 1:100 and 1:200. ***C***, Anti-DAT antibody SC-32258 displaying good signal in mouse tissue and no signal in rat tissue at a concentration of 1:100 and 1:200. ***D***, Anti-DAT antibody ZRB1525 displaying a good signal in mouse tissue at a concentration of 1:100 and 1:200, and a poor signal in rat tissue at a concentration of 1:200. ***E***, Anti-DAT antibody PT-22524-1-AP displaying weak signal in mouse tissue at concentrations of 1:100 and 1:200 and no signal in rat tissue at a concentration of 1:100 and 1:200. ***F***, Anti-DAT antibody D6944 showing good signal in mouse tissue at a concentration of 1:200 and a weak signal in rat tissue. ***G***, Anti-DAT antibody MA5-24796 showing good signal in mouse tissue and weak signal in rat tissue at concentrations of 1:100 and 1:200 ***H***, Anti-TH antibody in rat tissue used to demonstrate good detection of the 6-OHDA-lesioned side. Scale bar = 6 mm

Seven antibodies with immunogens for the DAT N terminus were tested using immunohistology (AB2231, MAB369, SC-32258, ZRB1525, PT-22524-1-AP, D6944, and MA5-24796). Antibody AB2231 showed no signal on the mouse tissue and had a very modest, barely detectable signal on the rat tissue at a concentration of 1:100 and 1:200 (Fig. 5*A*). Antibody MAB369 produced a moderate signal in WT mice but not in DKO mice at both concentrations of 1:100 and 1:200. MAB369 showed no signal in rat tissue (Fig. 5*B*). Antibody SC-32258 displayed a good signal in WT mouse tissue that was not present in DKO mouse tissue but produced no signal in rat tissue (Fig. 5*C*). Antibody ZRB1525 provided a good signal in WT mouse tissue that was not present in DKO mouse tissue at both 1:100 and 1:200 concentrations, and a good signal in rat tissue only at a concentration of 1:100 (Fig. 5*D*). Antibody PT-22524-1-AP showed a very weak signal in the WT mouse tissue and did not elicit a signal in the DKO mouse or rat tissue at both concentrations (Fig. 5*E*). Antibody D6944 produced a good signal in the WT mouse tissue but not the DKO mouse tissue at a concentration of 1:200 and did not produce a good signal in rat tissue (Fig. 5*F*). Antibody MA5-24796 elicited a good signal in WT mouse tissue and did not elicit a signal in DKO mouse tissue or rat tissue at concentrations of both 1:100 and 1:200 (Fig. 5*G*). Anti-TH antibody labeling in rat tissue was used as a control to demonstrate the 6-OHDA-lesioned side (Fig. 5*H*). Immunohistology results are scored (Table 3) and summarized in Table 4.

### High-resolution immunohistology for select N-terminal antibodies

**Figure 6.**
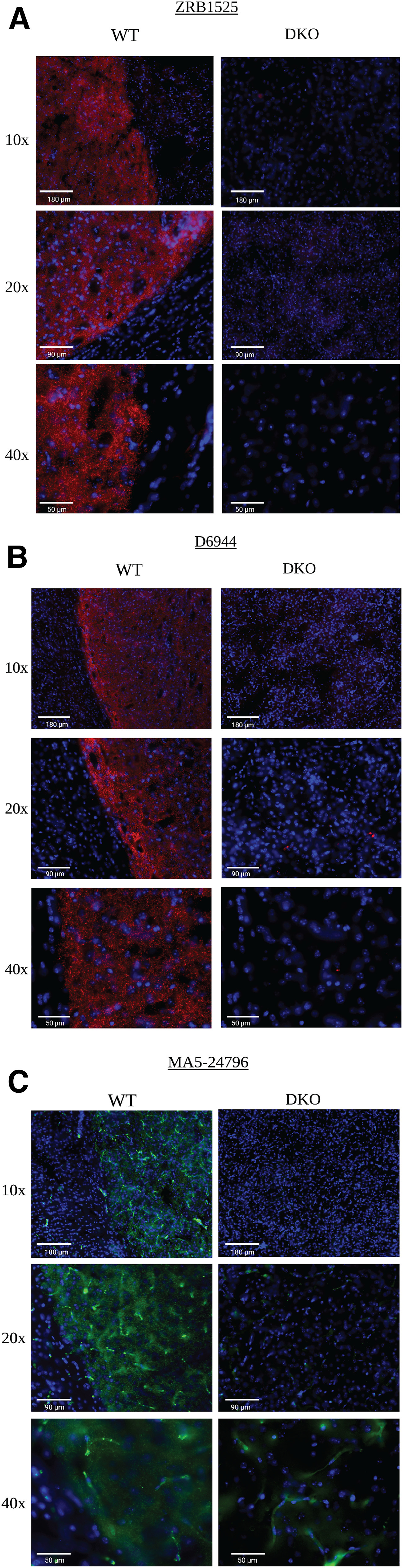
High-magnification immunohistology using antibodies with N-terminal immunogens. Immunohistology was performed using wild-type (WT) mouse tissue and DAT-knock-out (DKO) mouse tissue. All DAT antibody concentrations were performed at 1:200. DAPI signal is indicated in blue. ***A***, Anti-DAT antibody ZRB1525 (red) at magnifications of 10×, 20×, and 40×. Clear DAT signal differentiation is seen between WT and DKO with no nonspecific signal (no red signal). ***B***, anti-DAT antibody D6944 stained (red) at magnifications of 10×, 20×, and 40×. Clear DAT signal differentiation is seen WT with no signal in DKO. ***C***, Anti-DAT antibody MA5-24796 (green). Clear DAT signal differentiation is seen between WT and DKO with some nonspecific signal seen in DKO at 40× magnification. Scale bars are indicated in the bottom-left corner of each figure (white).

The three N-terminal antibodies (ZRB1525, D6944, and MA5-24796) were further evaluated in high-magnification immunohistology. Antibody ZRB1525 gave a good signal in WT but not DKO mice (Fig. 6*A*). Anybody, D6944, gave a good signal in WT but not DKO mice (Fig. 6*B*). Antibody MA5-24796, gave a good signal in WT, with some nonspecific signal in DKO mice because of the anti-mouse secondary antibody (Fig. 6*C*). Immunohistology results are scored (Table 3) and summarized in Table 4.

### Immunohistology using antibodies with DAT C-terminal immunogens

**Figure 7.**
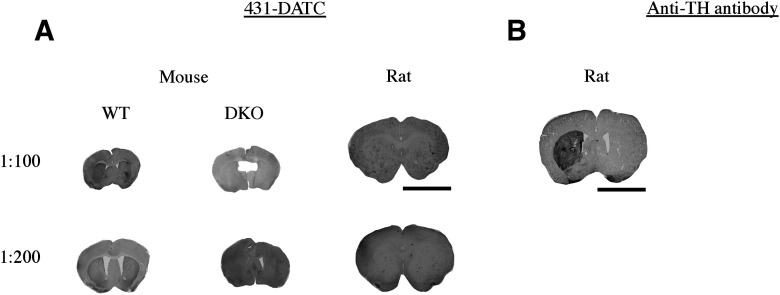
Immunohistology (IH) using an antibody with a DAT C-terminal immunogens. IH was performed using wild-type (WT) mouse tissue, DAT-knock-out (DKO) mouse tissue, or rat tissue in which one hemisphere was lesioned with 6-OHDA to kill dopamine neurons, thus eliminating the possibility of a DAT antibody to produce a signal on the lesioned side (internal negative control). Concentration of DAT antibody indicated in the figure. ***A***, Anti-DAT antibody 431-DATC displaying a faint signal in WT mouse tissue at a concentration of 1:100 and 1:200 and no signal in rat tissue at either concentration. ***B***, Anti-TH antibody in rat tissue used to demonstrate good detection of the 6-OHDA-lesioned side. Scale bar = 6 mm

One antibody (431-DATC) with an immunogen for the DAT C terminus was tested in immunohistology. The additional C-terminal antibodies, 600-401-C75 and SC-1433, are discontinued and unable to be purchased for experimentation and therefore were not further tested in histology. Antibody 431-DATC produced a very faint signal in WT mouse tissue that was not present in DKO mouse tissue and did not produce a signal in rat tissue (Fig. 7*A*). Anti-TH antibody labeling in rat tissue was used to demonstrate the 6-OHDA-lesioned side (Fig. 7*B*). Immunohistology results are scored (Table 3) and summarized in Table 4.

### Immunohistology using antibodies with DAT 2nd loop immunogens

**Figure 8.**
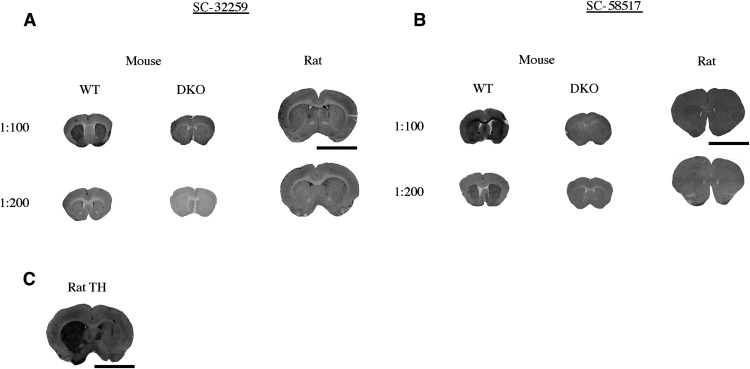
Immunohistology (IH) using antibodies with anti-DAT antibodies that have 2nd extracellular loop immunogens. IH was performed using wild-type (WT) mouse tissue, DAT-knock-out (DKO) mouse tissue, or rat tissue in which one hemisphere was lesioned with 6-OHDA to kill dopamine neurons, thus eliminating the possibility of a DAT antibody to produce a signal on the lesioned side (internal negative control). Concentration of DAT antibody indicated in the figure. ***A***, Anti-DAT antibody SC-32259 showing good signal in mouse tissue at a concentration of 1:100 but no signal in mouse or rat tissue at a concentration of 1:200. ***B***, Anti-DAT antibody SC-58517 with weak signal in mouse tissue at a concentration of 1:100 and 1:200 and no signal in rat tissue at 1:100 and 1:200. ***C***, Anti-TH antibody in rat tissue used to demonstrate good detection of the 6-OHDA-lesioned side. Scale bar = 6 mm

Two antibodies (SC-32259 and SC-58517) with immunogens for the DAT 2^nd^ loop were tested in immunohistology. Antibody SC-32259 provided a good signal in WT mouse tissue that was not detected in DKO mouse tissue, but only at a concentration of 1:100 and not at 1:200 (Fig. 8*A*). Antibody SC-32259 did not produce a DAT selective signal in rat tissue (Fig. 8*A*). Antibody SC-58517 produced a weak signal that was present in WT mouse tissue that was not present in DKO mouse tissue at both concentrations but did not produce a signal in rat tissue at either concentration (Fig. 8*B*). Anti-TH antibody labeling in rat tissue was used to demonstrate the 6-OHDA-lesioned side (Fig. 8*C*). Immunohistology results are scored (Table 3) and summarized in Table 4.

## Discussion

The goal of this manuscript was to evaluate various DAT antibodies commonly used in the literature and report on their ability and selectivity/specificity in detecting DAT in western blotting and immunohistology. The capability of a DAT antibody to perform well is essential, as adequate detection of DAT is necessary for a large range of scientific endeavors involving the dopamine system. Additionally, it is crucial that the detection of DAT does not differ dramatically across similar testing conditions within or outside of a given laboratory. Such drastic variation across different batches of DAT antibody can lead to difficulty in interpretation of results. Although the antibody MAB369 has been considered the “gold standard” in DAT detection, the lot of MAB369 tested in our studies (Fig. 1) did not provide strong detection of DAT in Western blottings. It is important to note that other studies have reported strong signals using MAB369 (Loder and Melikian, 2003; Sorkina et al., 2003; Torres et al., 2003a; Miranda et al., 2007; Boudanova et al., 2008a, b; Eriksen et al., 2009; Vina-Vilaseca and Sorkin, 2010; Vecchio et al., 2014). With LI-COR as our primary detection method used, it is possible that an adequate signal was not detected using MAB369 because of a lack of signal amplification. Indeed, most studies that report a strong signal with MAB369 use chemiluminescence approaches which offers strong amplification. Since our approach does not have a similar amplification as chemiluminescence, it could explain the lack of signal with MAB369 (lot #3188610) using LI-COR. Interestingly however, previous lots of MAB369 (lot #2610475) tested by our group provided good detection with our LI-COR imaging system as well (Medvedev et al., 2013; Beerepoot et al., 2016), which could indicate some batch-to-batch inconsistencies for this antibody.

With regards to other antibodies tested, it was found that two of the commonly used antibodies in the literature do not detect specific/selective DAT bands. Our results show that antibody AB2231 gave no specific DAT signal in western blotting and had extensive nonspecific binding in mouse striatal samples (Fig. 1). Similarly, antibody PT-22524-1-AP had no specific DAT signal in western blotting from brain tissue and gave a very weak and nonspecific signal in immunohistology (Figs. 2, 5). These two antibodies have been quite extensively used in the literature (Cone et al., 2013; McIntosh et al., 2013; Ruocco et al., 2014; Sconce et al., 2015; Hood et al., 2016; Calipari et al., 2017; Churchill et al., 2017; Y. Zhang et al., 2017; Alonso et al., 2021). Antibody AB2231 and PT-22524-1-AP use immunogens to the N-terminal regions of the rat dopamine transporter and human dopamine transporter, respectively. The specific sequences of their immunogens are not accessible. Although, in principle, these antibodies should produce a specific signal when binding to rat and mouse DAT in immunohistology and mouse DAT during western blotting, no specific signal was detected with these antibodies either in western blotting nor immunohistology assay. The mouse and rat DAT sequences are highly conserved; therefore, it is unlikely that lack of signal is because of species-specific detection issues. Furthermore, blocking procedures were performed with instrument specific (LI-COR) blocking buffer to prevent nonspecific binding, and primary antibody concentrations were prepared as per manufacturer recommendations for WB (Tables 2, 4). The simplest explanation might be that these antibodies are not adequate for detecting DAT.

Our western blotting and immunohistology results for PT-22524-1-AP antibody did not detect any specific DAT signal from animal tissue despite being performed as per manufacturer guidelines (Table 2). PT-22524-1-AP antibody immunogen sequence is based on an N-terminal region of the human dopamine transporter. The first 60 aa in the human DAT are more conserved in the mouse compared with the rat sequence (six variations in amino acid sequence compared with eight variations, respectively). This could potentially explain the weak signal detected in immunohistology of mouse tissue and no signal detected in the rat (Table 4). Interestingly, PT-22524-1-AP antibody did have good detection of human DAT protein expressed in HEK cells (Fig. 2*A*). Antibody ZRB1525’s immunogen also consists of an unspecified 18 amino acid portion of the human DAT N terminus. Interestingly, ZRB1525 produced a good signal in both mouse and rat brains in immunohistology, and a weak to moderate signal in western blotting from mouse tissue (Table 4). It can be speculated that the immunogen for ZRB1525 is in a region of the N terminus that is more similar among rats and mice compared with PT-22524-1-AP, resulting in better signal detection in mice and rats. Three antibodies (D6944, MA5-24 796, ZRB1525) were further evaluated in high-magnification immunohistology. These antibodies were chosen because they gave good signal detection in LI-COR immunohistology (Fig. 5*D*,*F*,*G*). By comparing WT and DKO samples stained for these antibodies, there is a clear strong signal in WT animals and a clear absence of signal in DKO slices, indicating that they also work very well at high magnification (Fig. 6). There was however some nonspecific binding seen at high magnifications with MA5-24796. We hypothesize that this is due the secondary anti-mouse antibody. Indeed, anti-mouse staining without any primary antibody showed green signal that was similar to background signal seen in DKO animals (data not shown), suggesting that the nonspecific signal in Figure 6*C* can be attributed to the specificity of the secondary antibody, and not the primary antibody.

Our results indicate that it is important and necessary to validate DAT antibodies using DAT-KO samples as a negative control to provide proof of specificity of a given DAT antibody. Of the numerous papers using various DAT antibodies for WB and/or immunohistology discussed and cited here (Loder and Melikian, 2003; Sorkina et al., 2003; Torres et al., 2003a; Sorkina et al., 2006; Miranda et al., 2007; Boudanova et al., 2008a, b; Lyck et al., 2008; Eriksen et al., 2009; Vina-Vilaseca and Sorkin, 2010; Cone et al., 2013; McIntosh et al., 2013; Ruocco et al., 2014; Gou et al., 2015; Sarkar et al., 2015; Sconce et al., 2015; Garea-Rodríguez et al., 2016; Hood et al., 2016; Churchill et al., 2017; Y. Zhang et al., 2017; Colon-Perez et al., 2018; Jovanovic et al., 2018; Perdikaris et al., 2018; Li et al., 2019; Rosas-Hernandez et al., 2019; Shah et al., 2019; Harraz et al., 2021; Niu et al., 2021; W. Zhang et al., 2021; Cuevas et al., 2022), only a handful used a DAT-KO sample as a comparator to determine specificity for DAT (Leo et al., 2018; Yang et al., 2019). Similarly, for immunohistology, use of DAT-KO brain slices or dopamine neuron lesions (e.g., 6-OHDA or MPTP) should be considered to ascertain antibody specificity/selectivity. We have summarized our western blotting and immunohistology results in Table 4 with a scoring system (Table 3) to simply represent the results for all the tested antibodies.

In summary, not all commercially available DAT antibodies detect DAT as advertised, and using such antibodies should be carefully considered in terms of application, specifics of protocol, and if the antibody has been tested against a proper negative control, such as a DAT-KO sample, to ensure selectivity and specificity.

## Synthesis

Reviewing Editor: Mikko Airavaara, Helsingin yliopisto Decisions are customarily a result of the Reviewing Editor and the peer reviewers coming together and discussing their recommendations until a consensus is reached. When revisions are invited, a fact-based synthesis statement explaining their decision and outlining what is needed to prepare a revision will be listed below. The following reviewer(s) agreed to reveal their identity: Hitoshi Inokawa, Miaomiao Mao.

In this work, the authors remind an important point of view for using an antibody. Researchers should be aware that even commercially established antibodies may not produce the expected results, depending on the lot, animal species and methodology such as western blot or immunohistochemistry. The authors claim that antibodies should be carefully considered in terms of application, specifics of protocol, and if the antibody has been tested against a proper negative control, such as a DKO sample, to ensure selectivity and specificity. This is one of the most fundamental aspects of scientific trials, but it is also something that has been neglected in recent years.

1. What is the rationale for using nude rats instead of SD rats. Because SD rats are more commonly used in experiments.

2. Since authors seem to have experience in obtaining good immunohistological results with MAB369, it would be more convincing to present those data.

3. Please review the text carefully and change u to μ.

4. Page 4 L161 LICOR should be LI-COR

5. Page 4 L179 Figure 1d should be Figure 1e. In addition to this, the figure 1d (ZRB-1525) should also be corrected.

6. Page 7 L275 LIC-COR should be LI-COR.

7. The Western blot protocol is not standardised and no explanations included for the choice of method.

8. Methodology is inadequately described. e.g. no specific details on equipment used, recovery time for 6-OHDA rat experiments, number of animals used for each experiment.

9. IHC images from the LICOR imaging have very poor resolution. For validation of antibodies, high magnification and high resolution confocal images should be included.

10. Discussion is insufficient and should include explanations for example, why some antibody dilutions did not work as expected or why most antibodies did not work in rats.

11. No methods description for HEK293 cell experiments.

12. Other minor points: No scale bars for IHC images, incorrect Figure 1d/e labelling, no use of secondary antibody controls for IHC, no housekeeping control for WB, inconsistency in describing results in text and table summary (see ZRB-1525).
